# Volar Locking Plate Breakage after Nonunion of a Distal Radius Osteotomy

**DOI:** 10.1155/2016/6836190

**Published:** 2016-11-28

**Authors:** Sergi Barrera-Ochoa, Sergi Rodríguez-Alabau, Andrea Sallent, Francisco Soldado, Xavier Mir

**Affiliations:** ^1^Hospital Universitari Vall Hebron, Orthopedic Surgery Department, Hand and Microsurgery Unit, Passeig Vall d'Hebron 119-129, 08035 Barcelona, Spain; ^2^Hospital Universitari Quiron-Dexeus, ICATME, Hand and Microsurgery Unit, C/Sabino de Arana 5-19, 08028 Barcelona, Spain; ^3^Vall Hebron Research Institute (VHIR), Musculoskeletal Tissue Engineering, Passeig Vall d'Hebron 119-129, 08035 Barcelona, Spain; ^4^Hospital Universitari Sant Joan de Deu, Pediatric Upper Extremity Surgery and Microsurgery Department, Barcelona, Spain

## Abstract

We report a 38-year-old male with a nonunion followed by plate breakage after volar plating of a distal radius osteotomy. Volar locking plates have added a new approach to the treatment of distal radius malunions, due to a lower morbidity of the surgical approach and the strength of the final construction, allowing early mobilization and return to function.* Conclusion*. Plate breakage is an uncommon complication of volar locking plate fixation. To our knowledge, few cases have been described after a distal radius fracture and no case has been described after a distal radius corrective osteotomy. In the present case, plate breakage appears to have occurred as a result of a combination of multiple factors as the large corrective lengthening osteotomy, the use of demineralized bone matrix instead of bone graft, and the inappropriate fixation technique as an unfilled screw on the osteotomy site, rather than the choice of plate.

## 1. Introduction

Plate breakage is an uncommon complication of volar locking plate (VLP) fixation. Very few case reports are currently available in the literature regarding plate breakage following a distal radius (DR) fracture [1–4] and no case is available after a DR corrective osteotomy. The present case is a 38-year-old male with a nonunion followed by plate breakage after volar plating of a DR osteotomy.

## 2. Case Presentation

**Figure 1:**
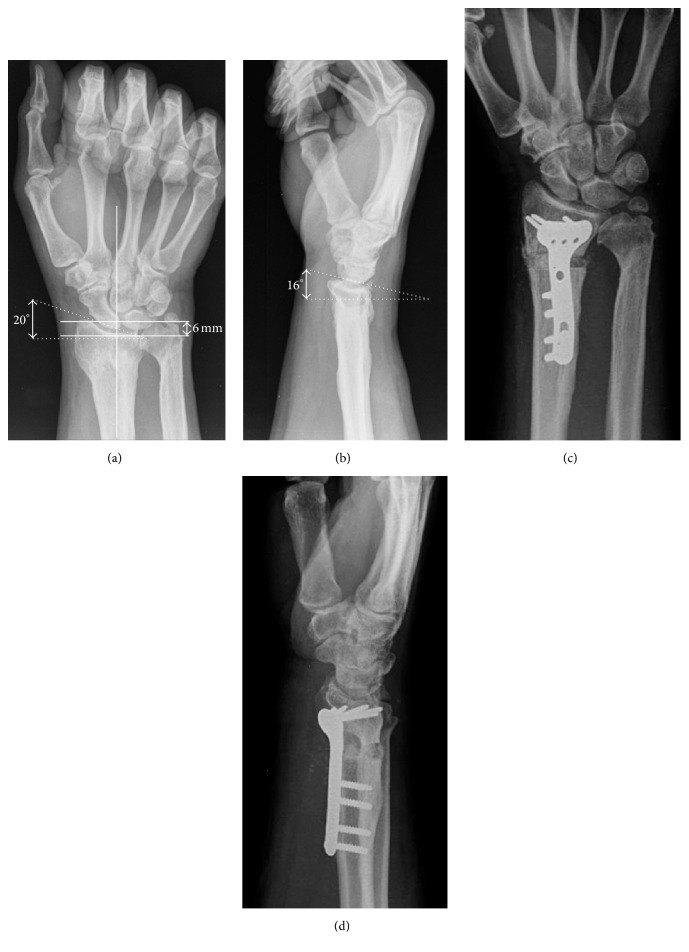
(a-b) Plain radiographs preoperative images showed DR malunion with a loss of volar tilt (−15°), radial inclination (20°), and positive ulnar variance (6 mm). (c-d) Postoperative radiographs showed DR osteotomy with a correction of volar tilt (2°), radial inclination (22°), and ulnar variance (0 mm).

**Figure 2:**
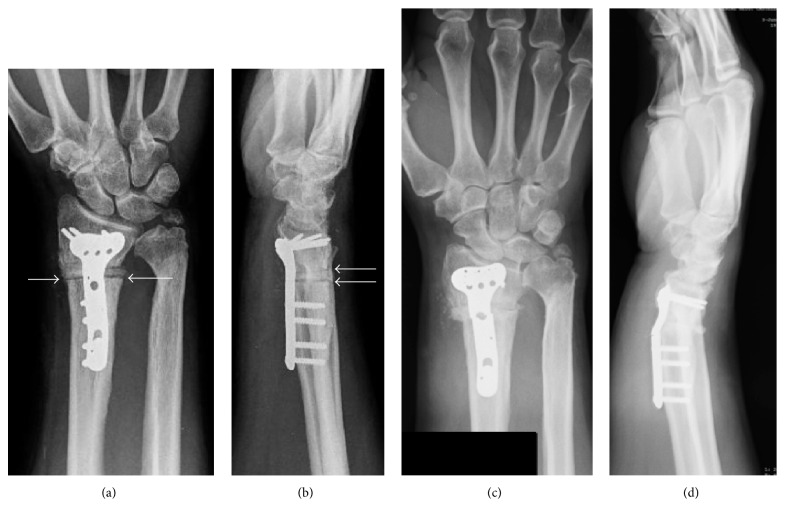
(a-b) Plain radiographs postoperative images at 3-month follow-up showed a development of radiolucent lines surrounding the graft at the osteotomy site (white arrows). (c-d) Plain radiographs postoperative images at 4-month follow-up showed plate breakage through the unfilled screw hole at the osteotomy site without plate bending or screw loosening.

A 38-year-old, right-handed male presented at our department with right wrist pain and difficulties in his job as a taxi driver, especially with wrist extension. He also referred to having pain in ulnar region of the wrist. He had no past medical history of interest. The patient had suffered right distal radius fracture twelve years before, conservatively treated with a cast for a six-week period. Plain radiographs and computed tomography images showed distal radius malunion with a loss of volar tilt and radial inclination and positive ulnar variance (Figure 1). Volar corrective osteotomy and internal fixation with a stainless steel volar locking plate (VLP) (Trimed Volar Bearing Plate™, Valencia, CA, USA) were performed (Figure 1). Distal to the osteotomy, three angle-stable locking screws were used on the radial side and one cancellous screw was placed on the ulnar side. One nonlocking cortical screws and three angle-stable locking screws were used proximally. The osteotomy site was grafted with demineralized bone matrix (Allomatrix, Wright Medical Corporation). The postoperative radiographs showed an improvement of the palmar tilt and radial inclination from −16° to 2° and 20° to 22°. Ulnar variance decreased from 6 mm to 0 mm. Postoperatively, the forearm was immobilized in a plaster cast for 3 weeks before starting rehabilitation. Two months after surgery, the splint was discontinued all day because wrist range of motion was complete (same ROM as the contralateral wrist) and pain-free, although progressive radiographs showed radiolucent lines surrounding the graft at the osteotomy site without plate bending or screw loosening. Four months after the operation, radiographs showed that the plate was broken through the unfilled screw hole at the osteotomy site and reduction was lost (Figure 2). Clinical examination revealed wrist tumefaction, shortening, and DR displacement. After ruling out infection, the patient was reoperated on, removing plate and screws. The nonunion was corrected and fixed with a longer stainless steel VLP (Trimed Volar Bearing Plate™, Valencia, CA, USA). Distal to the osteotomy, six angle-stable locking screws were used. One nonlocking cortical screws and six angle-stable locking screws were used proximally, filling all the proximal screw holes. The DR nonunion site was grafted with autologous iliac crest bone graft. The plaster cast was removed at six weeks and replaced by a removable splint, beginning physical therapy at that time. In addition, the material, including plate and screws, was sent back to the manufacturing company in order to analyze and detect any molecular material defect. None of these studies demonstrated anomalies in manufacturing process or metal composition.

**Figure 3:**
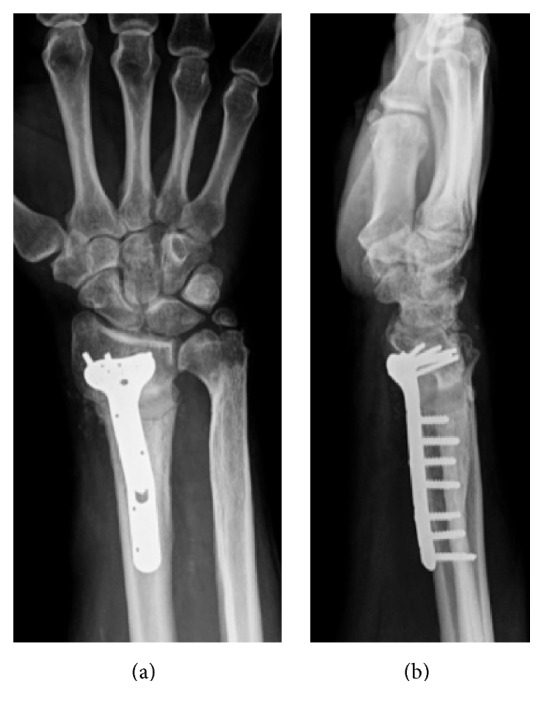
(a-b) Plain radiographs postoperative images at 8-month follow-up showed DR fixation with a longer stainless steel volar locking plate.

At the follow-up at eight months after the initial surgery, the fracture had consolidated clinically and radiologically (Figure 3). ROM was 60°–50° of flexion–extension and radial-ulnar of 15°–25°, all movements being completely painless. His grip strength was 80% of the opposite wrist and he restarted his job. One year after the last surgery, the patient remains painless and performs all activities.

## 3. Discussion

In recent times, VLP have added a new approach to the treatment of DR malunions, thanks to the low morbidity of the surgical approach and the strength of the final construction, allowing early mobilization and return to function [5–7]. Breakage of VLP is an uncommonly reported complication [1–7]. A review of over 350 patients with volar locking plate described 9 mechanical failures, including screw failure (7) and plate bending (2), but none with plate breakage [8].

De Baere et al. noted breakage of a 3.5 mm T-type™ titanium locking compression plate™ (LCP™) (Mathys Medical Ltd., Bettlach, Switzerland) three months after the fixation of a DR fracture [1]. Authors cited inadequate reduction and suboptimal contact between volar cortex and the plate contributed to increased load transmission through the implant and eventually caused plate breakage. Yukata et al. reported breakage of a Matrix Smartlock Titanium plate™ (Stryker Leibinger™, Freiburg, Germany), which had been implanted to stabilize an osteoporotic and multifragmented fracture [2]. They postulated that increased stress from early weight bearing was responsible for failure. Imade et al. have reported on a Matrix Titanium LCP (Stryker™, Kalamzoo, MI, USA), which broke only one week after surgery [3]. Authors hypothesized that placing the most distal screw in the proximal fragment too near to the fracture site accentuated the mechanical stress in that area, leading to plate failure. Khan and Gozzard reported breakage of 3.5 mm Titanium LC (Synthes, Solothurn, Switzerland) [4]. They suggested a multifactorial failure that included patient factors (noncompliance, smoking, and poor personal hygiene), biological factors (fracture fragmentation, impaired circulation, bruised muscular envelope, and secondary infection), and mechanical factors (unfilled screw holes and insufficient immobilization).

All these previous studies reported on cases of DR plate breakage that were fabricated from grade II titanium [1–4]. The titanium plates are more likely to fail than stainless steel plates [9]. The plates used in the current case were fabricated from 3.5 mm thick stainless steel. The anatomic design allows contouring to fit the volar surface of the distal radius. There are seven distal screw holes (2.4 mm) with threads that can accept either a locking or nonlocking screw and five proximal screw (3.5 mm) holes in the first plate and seven proximal screw holes in the second plate. We usually use plates longer than three proximal screw holes and fill all the distal row screw holes to fix distal radius osteotomies, because of the length of the plate and the distribution of the screws, both of which have been shown to be important factors determining the stability of fixation [9–11]. Biomechanical studies suggest that, for fractures of the radius, 3 or 4 screws should be used on each side of the fracture, because the forces acting on these bones are predominantly rotational [9–11].

The failure of the procedure in the presented osteotomy case is after a delayed union. Hardware failure during fracture healing has generally occurred after either a delayed union [1] or nonunion, although delayed union/nonunion of these osteotomies is very uncommon [5–7]. The possible causes of the delayed union in our case include biological factors (no bone graft on the osteotomy site) and mechanical factors (unfilled screw holes, large corrective lengthening osteotomy, and insufficient immobilization). We had not used bone graft according to some authors [5–7], who recommend that the volar cortex can be fixed directly with a VLP without structural bone grafting, even in severe deformities and osteoporotic bone. A segmental bone defect of 6 mm, where we could not fill the last diaphyseal screw, was created after the large corrective lengthening osteotomy. Plate breakages seem to occur in the vicinity of unfilled screw holes, adjacent to the fracture or osteotomy site [1–4, 11]. A locking mechanism on the screws implies that they cannot loosen out of the plate, increasing the load on the plate itself. If the biological environment is not conducive to fracture healing, the forces through the implant are exaggerated. Additionally, this high-stress area corresponds to the “bend” on these precontoured plates. It is corroborated by Trease et al.'s experiment, in which they axially loaded both locked and nonlocked plate constructs in osteotomy models and found that these always failed through the unfilled screw holes at the osteotomy site [11].

Both biological and biomechanical factors need to be considered when deciding on the type and mode of plate, the use and the type of bone graft, and the duration of immobilization. In our case, plate breakage appears to have occurred as a result of a combination of multiple factors as the large corrective lengthening osteotomy, the use of demineralized bone matrix instead of bone graft, and the inappropriate fixation technique (unfilled screw on the osteotomy site), rather than the choice of plate.
